# Efficiency of Novel Photocatalytic Coating and Consolidants for Protection of Valuable Mineral Substrates

**DOI:** 10.3390/ma12030521

**Published:** 2019-02-09

**Authors:** Andreja Pondelak, Sabina Kramar, Jonjaua Ranogajec, Luka Škrlep, Snežana Vucetic, Vilma Ducman, Andrijana Sever Škapin

**Affiliations:** 1 Slovenian National Building and Civil Engineering Institute, Dimičeva 12, 1000 Ljubljana, Slovenia; andreja.pondelak@zag.si (A.P.); luka.skrlep@zag.si (L.Š.); vilma.ducman@zag.si (V.D.); 2 Faculty of Technology, University of Novi Sad, Bul. cara Lazara 1, 21000 Novi Sad, Serbia; janjar@uns.ac.rs (J.R.); snezanap@uns.ac.rs (S.V.)

**Keywords:** photocatalytic coating, consolidant, protection, conservation, porous substrate, cultural heritage

## Abstract

In the process of protection and consolidation of valuable materials, the efficiency is the crucial property that needs to be considered. TiO_2_/ZnAl layered double hydroxide (LDH) coating and silicate- and carbonate-based consolidants were synthesized and proposed to be used for protection and consolidation of four porous mineral substrates: brick, stone, render and mortar. The photocatalytic efficiency of TiO_2_/ZnAl LDH coating, as well as consolidation efficiency of two consolidants, both applied on model substrates, were studied. The photocatalytic coating showed significant activity and performed well after the durability tests involving rinsing and freezing/thawing procedures. After treatment with both consolidants, a serious enhancement of consolidation of the used substrates was found. On the other hand, the application of TiO_2_/ZnAl LDH, as well as consolidants, caused negligible changes in the water vapour permeability values and in appearance of the porous mineral substrates, indicating a high level of compatibility.

## 1. Introduction

Over the last decade, the application of nanotechnology in the conservation of cultural heritage has aroused great interest, especially in the syntheses of consolidants and protective coatings. Consolidants based on calcium compounds have mainly been prepared as macro- and/or nanoparticles of calcium hydroxide in different alcohols [1,2,3,4]. The dispersion of calcium hydroxide nanoparticles in alcohol was shown to be an effective consolidating product for many types of works of art, especially wall paintings [5,6] and building stone [7,8]. The effectiveness of this type of consolidant is related to the small dimensions of the constituent particles and the ease of their penetration into the deteriorated material layers, as well as to an increased reactivity towards CO_2_ [6,7]. But there are still some drawbacks, such as variety of particle size, incomplete carbonisation and low concentrations of the active substance used. Moreover, a high concentration of active substance can enhance white haze and reduce the penetration of the consolidants into the substrate [4,7]. Consequently, in practice, due to low concentration of the active substance and volatility of solvents in the case of nano-lime consolidants, many applications are required for satisfactory consolidation [4,6,9]. For effective consolidation, solvents should exhibit a modest volatility, otherwise the penetration of consolidants based on Ca(OH)_2_ nanoparticles would be hampered. Namely, too dry an environment may cause fast evaporation of the alcohol used in the consolidant and prevent deep penetration, and may in some cases cause the formation of white haze. The use of soluble starting materials is desirable for effective consolidation of the deeper layers of the degraded substrates. However, the preparation of highly soluble, carbonate-based consolidant precursors is very difficult, as the solubility of calcium carbonate is low [3]. To overcome this problem, a water-based solution of calcium acetoacetate was synthesised and proposed for the consolidation of carbonate-based substrates [10]. As it is a water solution, which means lower evaporation rate of the solvent compared to organic solvents, deeper penetration into the material could be achieved, where a recohesion between particles could be established [11]. Moreover, due to higher concentrations of the consolidant, the number of successive applications of the consolidants can be significantly reduced, without any white haze on the treated surface [9].

Among the silicate consolidants, the most commonly used refer to alkoxysilanes, with the most frequent compound being tetraethyl orthosilicate (TEOS) [12,13], whose effectiveness derives from hydrolysis–condensation reactions. This leads to the formation of amorphous silica gel inside the stone pores [14]. The compatibility of the deposited silica gel with the silicate substrates and its ability to form strong Si–O–Si bonds are the main advantages that make the use of this product so common [15]. The temporary hydrophobicity of TEOS-treated substrates, the tendency of TEOS for crack formation during the drying process and the formation of dense fragments of gel inside the pores are the main limitations of this type of consolidant [13]. The silica gel that fills the pores could block them, resulting in the formation of a material that is less permeable to water vapour [16]. In order to overcome these limitations, several modifications have been introduced [17,18,19,20]. For silicate-based substrates, a modified formulation of a commercial product based on silicate ester was developed. Due to a balanced combination of polysilicate, dioxalane, a mixture of C11–C13 alkanes (liquid paraffin) and diethylethanolamine, the consolidant has a low dry mass [10] with uniform consolidation through the profile of the substrate [11].

Regarding the need for surface protection of the already consolidated materials, several attempts have focused on the development of self-cleaning and photocatalytically active materials [21]. The use of these advanced materials represents preventive and lasting surface protection, which is more effective than invasive and repeated actions. The photocatalytically active coatings for building materials allow the acceleration of deterioration of atmospheric pollutants, leading to their complete mineralisation and thus becoming harmless substances. The main attention of this research has focused on the application of nanosized TiO_2_ photocatalysts, due to their widespread availability, photocatalytic activity and high stability [22,23,24]. Since eventual inhalation of nanosized particles could present certain health hazards [25], the use of microporous TiO_2_ [26] or of properly immobilised TiO_2_ particles onto the photocatalyst support could eliminate these problems. One recent publication has presented the inorganic–inorganic nanocomposite photocatalysts based on inorganic minerals associated with TiO_2_. The layered-structure materials, such as anionic layered double hydroxides (LDHs), can tailor the physical and chemical characteristics of nanosized TiO_2_ during synthesis. They possess a large surface area and enable the absorption of organic substances on their external surfaces or within interlaminar spaces [27]. Besides the immobilisation of the TiO_2_ particles and the prevention of aggregation, LDH systems provide adequate porosity and compatibility with the mineral substrate [28]. As already mentioned, the photocatalytic activity of the TiO_2_ photocatalyst can be significantly enhanced by its immobilisation within a layered-structure material, producing a TiO_2_–inorganic nanocomposite [28]. Furthermore, a TiO_2_/Zn–Al nanocomposite powder with proven photocatalytic properties [29] could improve the compatibility between the photocatalytic coating and the substrate. Therefore, a nanocomposite photocatalyst based on a Zn–Al layered double hydroxide (ZnAl LDH) associated to TiO_2_ was developed [27]. The existing relation between the photocatalytic effect and the photo-induced hydrophilicy of the newly designed TiO_2_/LDH coatings, which is very important for sustaining the surface with self-cleaning properties, has already been reported [27]. Analysis of the photocatalytic coating applied on mineral surfaces of different mineralogy and porosity, considering the photocatalytic activity and durability, is still lacking.

Within this study, the efficiency of the silicate- and carbonate-based consolidants and the TiO_2_/ZnAl LDH coating was studied on four different porous mineral substrates (brick, stone, mortar, render) which was performed in the frame of the 7th FP HEROMAT project [30]. The consolidated samples were characterised by means of spectrophotometry, resistance to abrasion, DRMS, Hg-porosimetry gas sorption and water vapour permeability. The performed study also provides an insight into the photocatalytic activity, before and after the rinsing and freeze–thaw treatment of mineral substrates with a TiO_2_/ZnAl LDH coating.

## 2. Materials and Methods

### 2.1. Model Substrates

For selection of model substrates, we focused on the imitation of real construction materials from two historical sites, namely Bač Fortress (Serbia) and Dornava Manor (Slovenia), considered in the scope of the HEROMAT project [30]. Several types of bricks and mortars, as well as stone elements and facade surfaces covered with a render, have been found in the construction of Bač Fortress and at Dornava Manor. The substrates were specified and analysed in detail in an earlier investigation [31]. The following model substrates were selected and prepared for the present study: (i) carbonate brick (designated as brick), (ii) sandstone (designated as stone), (iii) lime mortar with a carbonate aggregate (designated as mortar) and (iv) render substrate (designated as render).

### 2.2. Materials and Application on the Substrates

#### 2.2.1. Consolidants

For silicate substrates (brick, stone), a new silicate-based consolidant formulation (designated as CF4) was prepared by mixing 50 wt.% ethyl polysilicate (WACKER TES 40 WN, Wacker Silicones, WACKER, Budapest, Hungary), 20 wt.% solvent (1,3-dioxolane – DOX, 99%, Sigma-Aldrich), 5 wt.% catalyst (diethylethanolamine – DEEA, 99.5% Sigma-Aldrich) and 25 wt.% the mixture of C11–C13 alkanes (paraffin, Samson Kamnik, CAS: 64742-48-9). The catalysts were always mixed into ethanol and added last to the rest of the mixture. The role of addition of the mixture of C11–C13 alkanes has been described in detail elsewhere [11]. The consolidation action of silicate-based consolidant is based on the sol–gel process and occurs through hydrolysis and a water and/or alcoholic condensation reaction which leads to the formation of silica gel [32].

For carbonate substrates (mortar, render), a water-based consolidant formulation of calcium acetoacetate (Ca(OAcAc)_2_) (designated as CFW) was developed, which was thoroughly described in the patent [10]. In the research, 9.6 wt.% Ca(OAcAc)_2_ was used (corresponding to 4% of theoretically formed CaCO_3_). Additionally, a catalyst (0.05 wt % ethylenediamine, 99+%, Acros Chemicals) was added into the consolidant before application. The principle of consolidation with calcium acetoacetate consolidant is as follows: calcium acetoacetate in the presence of water (moisture in the air or from the solution) decomposes into calcium carbonate, carbon dioxide and acetone [10].

Both consolidants were applied directly onto the upper surface of the substrate using a pipette. The quantities of the consolidant applied per individual model were as follows: 0.05 g/cm^2^ of CF4, 0.1 g/cm^2^ of CF4, 0.1 g/cm^2^ of CFW and 0.02 g/cm^2^ of CFW onto stone, brick mortar and render, respectively. The exact concentration of ingredients for CFW and CF4 formulations are described in previous paragraphs. Samples were exposed for four weeks at 23 °C and 50% relative humidity and then analysed by different techniques.

#### 2.2.2. Photocatalytic Coating

For the synthesis and development of the protective photocatalytic coating formulation (designated as PF) with self-cleaning properties, inorganic–inorganic nanocomposites based on LDHs associated with a photocatalytically active TiO_2_ were synthesised. The synthesis parameters (temperature, pH, ageing time, etc.) were changed accordingly to the desired functional properties (photocatalytic activity, surface properties, particle size distribution, phase composition, durability, etc.). The commercially available Zn and Al precursors were continuously added together with an alkaline solution in order to maintain a constant pH value (9–9.5) during the synthesis. A low-supersaturation coprecipitation method was used in order to synthesise the TiO_2_/LDH nanocomposite [27]. The amount of the photocatalytic active TiO_2_ intercalated into the LDH structure was up to 10 wt.% [33]. After dilution of the synthesised concentrate and the stabilisation procedure, a photocatalytic suspension was prepared. It was applied by spray technique directly onto the model substrates. The procedure of application of the photocatalytic suspension was as follows: the pressure of the compressed air during the application was 6 bar; the hole in the nozzle had a diameter of 0.6 mm; the total number of deposited layers was three; the period between two applications was 5–10 min. After the application of the suspension in three layers, the treated substrates were dried at room temperature. After four weeks of drying, the coated samples were analysed by different techniques.

### 2.3. Methods

Spectrophotometry was performed in order to quantify the influence of the newly developed consolidants and coating on the colour difference and aesthetic appearance of the chosen mineral substrates. colorimetric parameters were measured with a spectrophotometer (Konica Minolta CM-2500c, Konica Minolta, Osaka, Japan), using the Lab colour space (Commission Internationale de I‘Eclairage [CIE] 1976), with the following characteristics and operating conditions: D65 standard illuminant and 10° observer. From CIE L^*^a^*^b^*^ colour values, the total colour differences before and after application of two selected consolidants or the coating precursor were calculated as follows [34]:

∆*E*_ab_^*^ = (∆L^*2^ + ∆a^*2^ + ∆b^*2^)^1/2^ (1)

In addition, the values ∆L^*^, ∆a^*^ and ∆b^*^ were calculated from the formulas: ∆L^*^ = L^*^_sample_ – L^*^_standard_; ∆a^*^ = a^*^_sample_ – a^*^_standard_; and ∆b^*^ = b^*^_sample_ – b^*^_standard_, respectively, where the sample values are those after the treatment, while the standard refers to values before the treatment. A positive value of ∆L* means that the sample is lighter than the standard, while a negative value of ∆L^*^ indicates that the sample is darker than the standard; +∆a^*^ and +∆b^*^ mean that the sample is redder or yellower than the standard, respectively, while –∆a^*^ and –∆b^*^ indicate that the sample is greener or bluer than the standard, respectively [35]. The results are expressed as the mean values of five measurements for each selected substrate.

In order to assess the consolidation effect, abrasion resistance was determined on samples with diameter of 12 cm by measuring the length of the recess obtained by a rotating and abrasive steel plate, according to EN ISO 10545-6/2012 [36]. The test specimen was placed in the apparatus so that it was tangential to the rotating disc (velocity of rotation was 75 rpm) and abrasive white fused Al_2_O_3_, grain size F80, was dropped into the contact between the rotating disc and the specimen at a rate of 100 g per 100 revolutions. The chord length of the groove left by the abrasive material was measured after 50 revolutions of the disc. Three measurements were performed for each treated and nontreated substrate.

Drilling resistance was used on samples with diameter of 12 cm and thickness of 2 cm to quantify the efficiency of the consolidant through the profile of the substrate. The microdrilling test was performed on the DRMS Cordless (SINT Technology, Florence, Italy). A 3 mm flat drill bit was used and a rotation speed of 1000 rpm and penetration rate of 5 mm/min were selected for the stone and render substrates. A 5 mm drill bit was used and a rotation speed of 400 rpm and penetration rate of 40 mm/min were used for the brick and mortar substrates. The results are expressed as the mean values of the three measurements of drilling force along the total length of the hole.

The pore system of the model substrates before and after the application of the consolidants was investigated by means of mercury intrusion porosimetry (MIP). Small representative fragments (overall depth of approximately 1 cm), approximately 1 cm^3^ in size, were dried under low pressure for 24 h and then analysed by Micromeritics® Autopore IV 9500 equipment (Micromeritics, Norcross, GA, USA). The substrates were analysed within the range of 0 to 414 MPa using penetrometers for solid substrates.

Nitrogen adsorption measurements were performed at 77 K using a Micromeritics ASAP-2020 analyser (Micromeritics, Norcross, GA, USA). The total specific surface area, the total pore volume and the pore-size distribution curves of the substrates were determined using the BET (Brunauer–Emmet–Teller) method, t-plot analyses and the BJH (Barret–Joyner–Halenda) methods, respectively. Two measurements were performed for each, treated or nontreated, substrate.

The water vapour permeability test was performed on two selected model substrates: brick (representing silicate-based substrate) and mortar (representing carbonate-based substrate) according to EN ISO 12572:2002 (wet conditions) [37]. Measurements were performed on model substrates with diameter of 12 cm after separate application of the consolidants and the photocatalytic coating. The assessment of water vapour permeability was evaluated by determining the water vapour diffusion resistance coefficient (vapour flux (WVF – μm/A) of the substrates before (blank) and after the application of the consolidant or photocatalytic coating.

The photocatalytic activity of the coated substrates (4 cm × 4 cm × 1 cm) was measured in the liquid phase by monitoring the change of rhodamine B (RhB) concentration under UV–vis irradiation [38]. The RhB presents a dye pollutant in which decomposition starts in the presence of light energy and a photocatalyst [39,40].

For the characterization of the newly developed photocatalytic material, two kinds of investigations were performed. Namely, mineralogical (XRD) analysis was done by Diffractometer PW 1729 CuKα (Philips, Amsterdam, Netherlands), XRD Philips device and morphological investigations by Scanning Electron Microscopy (SEM analysis)-JEOL JSM 6460 LV (JEOL Ltd, Tokyo, Japan).

In order to saturate the substrates before the photocatalytic assessment, a preabsorption test (24 h) with RhB solution was carried out. After the preabsorption procedure, the used RhB solution was replaced with a fresh one. The substrates were irradiated for 30 min, 90 min, 150 min, 210 min and 24 h (OSRAM EVERSUN lamp model L 40–79 K, intensities of UV-A and visible light spectra were 0.8 mW/cm^2^ and 0.3 W/m^2^, respectively). A UV–vis spectrophotometer (Evolution 600 spectrophotometer, Evolution 600 spectrophotometer, Thermo Fischer Scientific Inc, Madison, WI, USA) was used to monitor the change in RhB concentration at the main absorption peak (λ = 554 nm). The photocatalytic activity was evaluated based on the RhB removal efficiency and is expressed by the following equation [39]:

A(%) = [(*c_0_* − *c*)/*c_0_* ]·100 (2)

where *c_0_* is the RhB concentration of the treated substrate in the dark at a defined time and *c* is the RhB concentration of the substrate under UV–vis light at a defined time.

In order to assess the stability and durability of the new materials after application to the chosen model substrate, the photocatalytic activity of the developed systems (coated substrates) was examined after the rinsing and freezing–thawing cycles, which simulate severe outside conditions.

A rinsing procedure was conducted on the brick, stone, mortar and render substrates treated with photocatalytic coating. A specific device was designed in accordance with the literature [41], which provided a constant flow of tap water (250 mL/min) through a pipe system (nozzle diameter of 0.90 mm). The water streams fell onto the coated substrates for 30 min and after that period the photocatalytic activity was measured. The obtained results were compared with those acquired before the rinsing procedure.

The freeze–thaw cycling was performed according to EN 12371:2010 [42]. The substrates of size 5 cm × 5 cm × 1 cm, treated with the photocatalytic coating (coated brick, stone, mortar and render; protected brick), were exposed to 50 cycles of freezing–thawing. One cycle consisted of 5.5 h at 15 °C in water, then the water was sucked out of the chamber and the temperature reduced to −4 °C within 2 h, then over the next 4 h the substrates were cooled to −10 °C, and after that the water was poured into the chamber again.

## 3. Results and Discussion

### 3.1. Consolidants

One of the performance requirements for consolidants is the appearance of the consolidated historic material, which should be as close as possible to the original. The results of measuring the colour differences of selected substrates after treatment are presented in Table 1.

Due to the fact that a white haze can be formed after the treatment with nano-based consolidants [4], it seems that the most important contribution to colour variation after the treatment comes from the total colour difference (∆E^*^), followed by the changes in luminosity (∆L^*^). Based on the obtained colour differences after the application of the new consolidants onto the porous mineral substrates, the identified compatibility criteria [43] can be underlined as low (∆E^*^ < 3) for the majority of model substrates, indicating low risk of chromatic incompatibility between the nontreated and treated substrates. Only in the case of brick did the obtained colour difference after consolidation reach 3.7, which is considered as a medium compatibility criterion (3 < ∆E^*^ < 5). Generally, the obtained colour difference may refer to a darkening of the surface after the treatment with silicate consolidant (−∆L^*^), while in the case of carbonate consolidant, the colour difference of the substrates after treatment is due mainly to slight yellowing (+∆b^*^) and darkening (−∆L^*^) effects. These findings indicate that newly developed consolidant CFW showed no whitening effect on the surface after consolidation.

The value of abrasion resistance, indicating the efficiency of the consolidation, was also determined by measuring the length of the recess and the volume of removed material. As shown in Table 2, the length of the obtained recess generally decreased after consolidation, indicating greater hardness of the treated substrates. The difference was especially significant in the case of brick (treated with CF4) and mortar (treated with CFW) substrates, where the length of recess (L) decreased after treatment from 52.0 to 46.8 mm and from 83.3 to 68.3 mm, respectively. The same trend is observed in the case of the volume of the removed material, which was reduced for the consolidated substrates, and reduction was higher for brick and mortar (from 1202 to 868 mm^3^ and from 5179 to 2748 mm^3^ for brick and mortar substrates, respectively). These two substrates were found to be much softer then stone and render (Vickers microhardness of brick and mortar were 13.2 HV and 20.1 HV, respectively, while stone and render had higher hardness: 66.7 HV and 40.1 HV, respectively).

**Figure 1:**
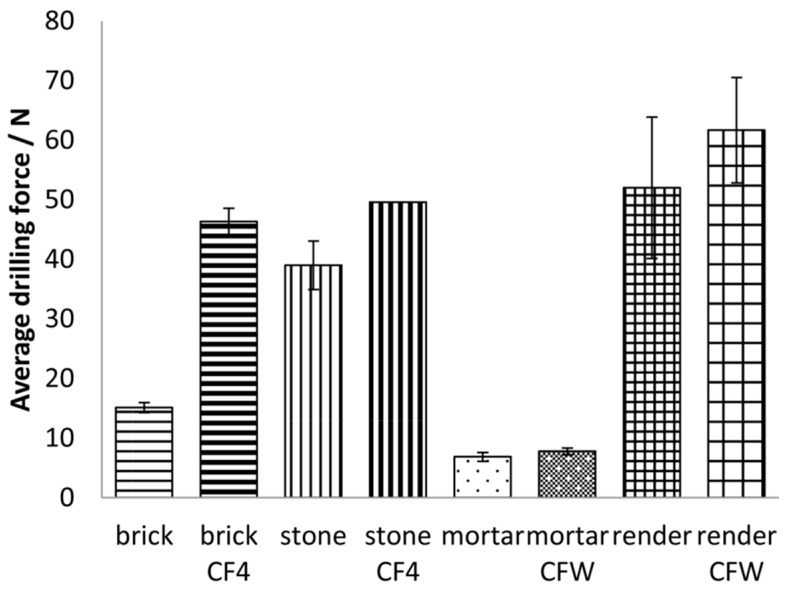
Average drilling resistance values of different substrates before and after the application of the two consolidants.

**Figure 2:**
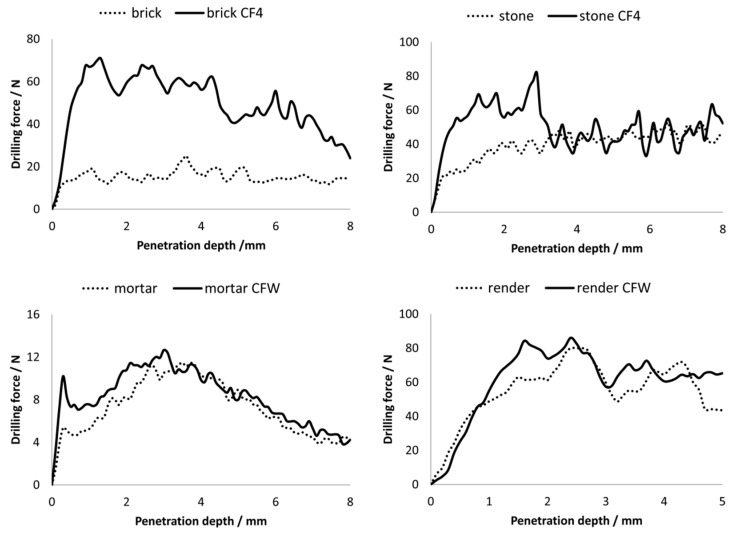
Average force vs depth graph of the substrates before and after the application of the two consolidants (the results are mean values of three measurements).

The results of measuring the resistance to abrasion were further confirmed by the DRMS method (Figure 1 and Figure 2). The average drilling force for each consolidated substrate, before and after application of the two consolidants, is presented in Figure 1, while the average forces of three measurements as a function of depth of the substrates before and after the application of the consolidants are shown in Figure 2. The highest increase in average drilling force (related to increase in mechanical strength) was observed in the brick sample after consolidation with silicate-based consolidant; the drilling forces increased from 15.1 N to 46.3 N. A significant increase in average drilling force was also found in the case of the stone substrate: the force increased from 39.0 N to 49.6 N. On the other hand, for the carbonate-based consolidant, smaller increase in force was observed: from 6.9 N to 7.8 N after the consolidation of the mortar substrate and from 52.0 N to 61.7 N after consolidation of the render substrate. Due to the nature of the consolidant, a greater increase in these values was expected for the substrates treated with the silicate consolidant. We found out that there is a close relation between consolidation efficiency of consolidants and porosity of treated substrates. For this reason, the porosity of substrates was thoroughly investigated and these results are described in detail hereafter.

As seen from Figure 2, higher penetration depths were achieved in the case of more porous substrates: for brick with 44.2% porosity (Table 3), the penetration depth was obtained through the entire observed depth (8 mm), while in the case of less porous (12.3%) silicate-based stone substrate, the penetration depth was observed up to 3 mm. For substrates treated with the carbonate-based consolidant, the average drilling forces after the consolidation were not significantly higher with respect to nontreated substrates; however, the enhancement was quite constant throughout the drilling depth. The pronounced higher consolidant effect (higher increase in drilling forces) in the case of substrates consolidated with silicate-based consolidant could be explained by strong Si–O–Si bonds, while in the case of carbonate-based consolidant, bonds formed between CaCO_3_ particles and substrate were weaker.

**Figure 3:**
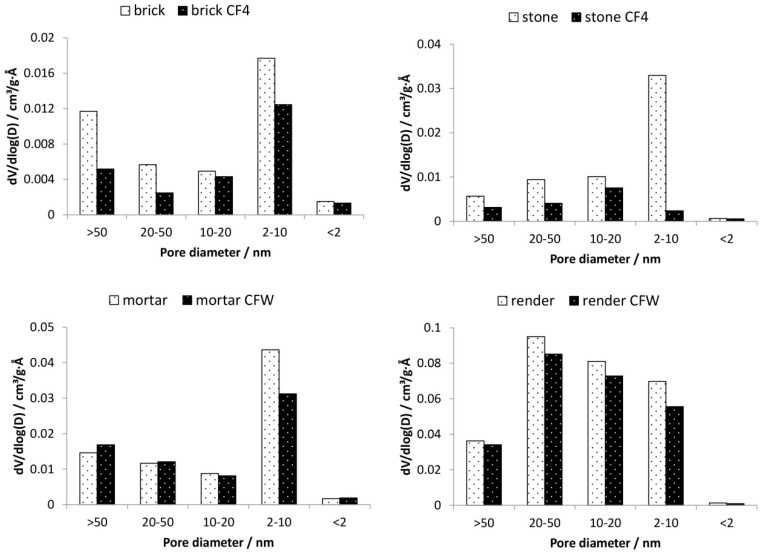
Results of Hg-porosimetry. Pore-size distribution of the mineral substrates before and after the application of the consolidants (CF4—silicate-based consolidant, CFW—carbonate-based consolidant).

**Figure 4:**
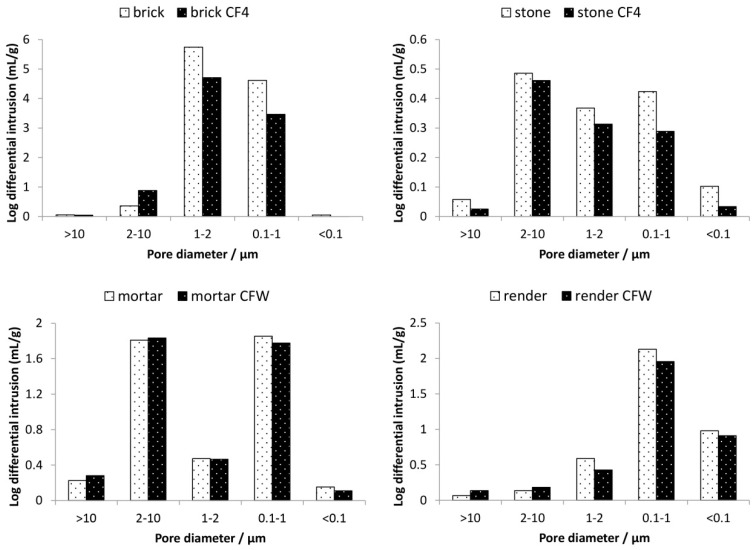
Results of gas sorption. Pore-size distribution of the mineral substrates before and after the application of the consolidants (CFW—carbonate-based consolidant, CF4—silicate-based consolidant).

Microstructural alterations induced by the two consolidants were examined in terms of porosity and BET surface area. Table 3 shows the parameters determined by MIP (porosity) and gas sorption (BET surface area), whereas Figure 3 and Figure 4 show the pore-size distribution before and after the treatment with the two consolidants obtained by MIP and gas sorption, respectively.

The silicate-based consolidant (CF4) reduced the porosity of the treated substrates (decrease in porosity after treatment was from 44.2% to 40.2% and from 12.3% to 9.8% for brick and stone substrates, respectively—Table 3), indicating that the consolidant caused a partial closing of the pores in the substrates. In terms of pore-size distribution (Figure 3), CF4 resulted in a decrease in the percentage of pores, which is especially evident for the stone substrate. In the case of brick, a slight increase in pores with radius 2–10 μm was observed, probably due to the merging of some mesopores into macropores. The modification of the pore system of the substrate with CFW was much less. The porosity of the substrates treated with the carbonate-based consolidant did not change significantly and remained constant before and after treatment (Table 3). However, the deposition of the consolidant into the pores resulted in a slight decrease in pores smaller than 2 μm (ranges 1–2 μm, 0.1–1 μm, < 0.1 μm), which is observed in the case of both substrates treated with CFW. On the other hand, in the ranges 2–10 μm and > 10 μm, a slight increase in pores was observed.

The BET surface area of the substrate consolidated with CF4 was also slightly reduced. As seen from the gas sorption results (Figure 4), the silicate-based consolidant reduced also the pores in the range < 50 nm. A similar trend was observed also for CFW-treated substrates. The decrease in the specific surface area indicates that fine pores were reduced for substrates treated by both consolidants. This is beneficial, as the increase in the fraction of smaller pores is known to raise the susceptibility of porous materials to decay due to the higher pressure caused by salt crystallisation. Consequently, treated materials could be considered as more prone to mechanical deterioration.

Results showed a clear reduction in macropores (r > 50 nm) and mesopores (2 nm < r < 50 nm). Since there was no increase in micropores (r < 2 nm) for substrates consolidated with the two consolidants, this indicates that the consolidant itself does not contribute to the microporosity. The reduction in porosity, as well as pore-size radius, was higher for CF4. Considering that the consolidants are usually required to alter the microstructure of materials to the lowest possible extent [43], and in particular, to cause minimum increase in smaller pores (i.e., pores having radius < 1 μm), both consolidants can be considered as compatible in terms of alteration of porosity.

In the case of stone, render and mortar substrates, it needs to be emphasized that the results of decrease of porosity, pore size distribution and BET surface area were obtained as average values of representative fragments (along the 10 mm thickness), while the consolidation of all three substrates took place at only approximately 3 mm in depth and so it is expected that all changes in distribution of pores occurred in this thin layer. This signifies an important limitation in interpreting the results of porosity, pore size distribution and gas sorption. Based on the obtained results of water vapour permeability (Table 4), it was concluded that the application of the two consolidants to the chosen mineral substrates did not change the diffusion resistance factor (µ) compared to the nontreated substrates. This suggests that the two consolidants do not block the surface pores, as these substrates appear to be almost identical, indicating no change in permeability between the treated and the nontreated substrates. This is beneficial, as the consolidant should allow water vapour transmission in order to avoid the accumulation of moisture and, consequently, soluble salts behind the treated material [44], which would lead to enhanced deterioration in this zone. Some authors reported that some treatments could cause reductions in permeability, even as high as 30% [45].

### 3.2. Photocatalytic Coating

**Figure 5:**
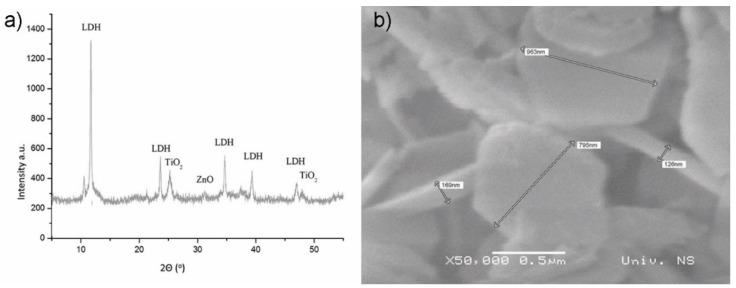
Characterization of newly developed photocatalytic coating: (**a**) phase composition (XRD analysis and (**b**) morphology analysis (SEM analysis).

Based on the XRD results shown in Figure 5a, the presence of the layered double hydroxide crystal structure (LDH) and titania was identified in the newly developed photocatalytic coating. The presence of ZnO phase was also confirmed by XRD analysis. The successful synthesis of the LDH material was additionally confirmed by the analysis of its morphology (SEM analysis, Figure 5b). The presence of the typical hexagonal plate-like particles was identified.

The values of the total colour difference after the application of the photocatalytic coating by a spray technique are minor and are identified as low (∆E^*^ < 3) (Table 1). According to compatibility criteria listed in the literature [43], the obtained colour difference indicates a low risk of chromatic incompatibility before and after the application of the photocatalytic coating. Moreover, the results show no whitening or yellowing after the application of the coating, which is shown as almost zero ∆L^*^ value in the case of all treated substrates. The results clearly evidence a high level of compatibility between the newly designed coating and the mineral substrates used.

Water vapour permeability was examined for the substrates with the highest total porosities (brick and mortar substrates) (Table 4). The application of the photocatalytic suspension on the surface of these substrates led to negligible changes in the water vapour permeability values in comparison with the nontreated substrates.

**Figure 6:**
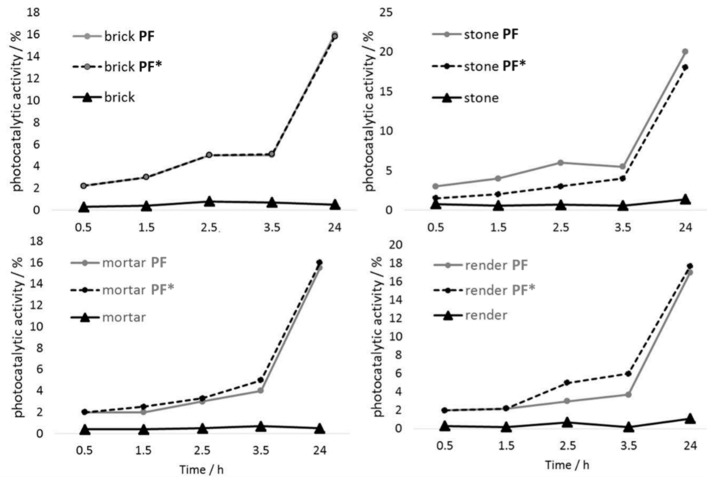
The photocatalytic activity of the coated mineral substrates before (PF) and after (PF*) the rinsing procedure.

Based on the obtained results, shown in Figure 6, the effect of photocatalytic degradation of the Rhodamine B is evident, even in the first 30 min of the experiments. Moreover, in order to assess the durability of the developed coating on the examined mineral substrates, the photocatalytic activity values before and after the rinsing test were examined (Figure 6). Evidently, no decrease in the photocatalytic values was seen after the applied rinsing procedure. Based on these results, it can be concluded that the developed and applied suspension is a photocatalytic material suitable for application onto mineral building materials.

**Figure 7:**
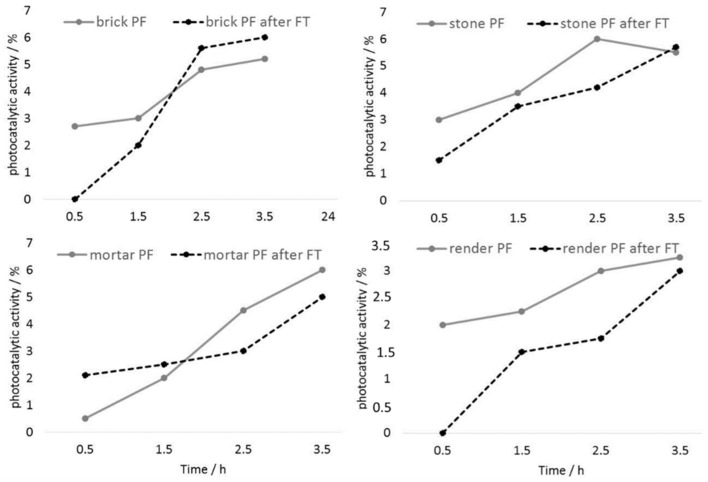
Photocatalytic activity of the treated mineral substrates before (PF) and after the freezing–thawing procedure (PF after FT).

In order to assess the stability of the photocatalytic coating to fluctuations of temperature, the values of activity of the photocatalytic coating were examined after freezing–thawing cycles (Figure 7). In the case of brick substrates, due to the presence of carbonate grains, some deterioration in the bulk of the substrates was identified, although the photocatalytic coating showed no significant changes (Figure 7). In the case of the mortar substrates, the cycles of freezing and thawing had a small but positive effect on the photocatalytic activity: an increase in this value was identified, probably as a consequence of the TiO_2_/LDH structure opening due to additional TiO_2_ action. Regarding the photocatalytic activity assessment of the stone and render substrates, no changes in photocatalytic activity were identified. Evidently, it can be concluded that the applied coating is sufficiently durable under the freezing–thawing cycles used.

## 4. Conclusions

The present work focused on the characterisation of the effectiveness of the treatment of four different mineral substrates with synthesised carbonate-based and silicate-based consolidants and photocatalytic coating.

The main conclusions are summarised as follows:

● In the case of silicate-based consolidant, generally a low risk of chromatic incompatibility (∆E^*^ < 3) between the nontreated and treated substrates was shown. We found a decrease in the volume of removed material (increased abrasion resistance) after treatment, as well as an increase in the DRMS resistance through the profile of the substrates, indicating greater hardness of the treated substrates. After consolidation, the substrates showed a decrease of porosity of the consolidated substrate, while water vapour permeability did not change;
● Treatment with a carbonate-based consolidant showed no whitening of the surface, with low risk of chromatic incompatibility (∆E^*^ < 3) between the nontreated and the treated mineral substrates. The study showed enhancement of consolidation after treatment, by a decrease in the volume of removed material after consolidation and by a small increase in DRMS resistance. Carbonate-based consolidation had a negligible effect on the pore structure of the mineral substrates, as well as on their water vapour permeability;
● The newly designed photocatalytic suspension based on TiO_2_/LDH showed negligible changes in the water vapour permeability and colour change values compared to the nontreated substrates. Besides good compatibility, the obtained results indicate good durability of the developed protective TiO_2_/LDH coatings, as well as a strong impact on the photocatalytic properties of the porous building materials, even after the durability tests involving rinsing and freezing/thawing procedures.

The presented study shows promising performances of the two consolidants and the photocatalytic coating developed within the HEROMAT project for the application to different porous mineral substrates.

## Figures and Tables

**Table 1:** Colour difference of the surface of the substrates before and after the application of developed consolidants (CF4, CFW) or photocatalytic coating (PF).

Substrate Treated with Consolidant or Coating	∆L*	∆a*	∆b*	∆E^*^
brick CF4	−3.63	+0.55	+0.65	3.7
stone CF4	−2.9	+0.13	+0.23	2.9
mortar CFW	−1.27	−0.34	+2.63	2.9
render CFW	−1.16	+0.56	+2.33	2.7
brick PF	−0.09	−0.01	−0.80	1.0
stone PF	+0.21	−0.15	+0.23	0.8
mortar PF	−0.36	−0.30	+0.49	1.1
render PF	−0.21	−0.15	+0.23	0.6

**Table 2:** Results of resistance to abrasion test.

Substrate with or without Consolidant	Length L (mm)	Volume of the Removed Material V (mm^3^)
brick	52.0 ± 2.0	1202 ± 140
brick CF4	46.8 ± 1.3	868 ± 70
stone	34.8 ± 0.4	353 ± 8
stone CF4	34.0 ± 0.5	335 ± 15
mortar	83.8 ± 3.8	5179 ± 698
mortar CFW	68.3 ± 0.3	2748 ± 31
render	25.3 ± 0.3	135 ± 12
render CFW	23.5 ± 0.5	109 ± 7

**Table 3:** Mercury intrusion porosimetry (MIP) and BET surface area of the substrates with and without consolidant.

Substrate with and without Consolidant	Brick	Brick CF4	Stone	Stone CF4	Mortar	Mortar CFW	Render	Render CFW
Porosity (%)	44.2 ± 0.2	40.2 ± 0.1	12.3 ± 0.2	9.8 ± 0.7	30.2 ± 0.5	30.8 ± 0.2	26.7 ± 0.2	26.0 ± 0.4	
BET surface area (m^2^/g)	1.26 ± 0.0	0.97 ± 0.1	1.99 ± 0.1	0.36 ± 0.0	2.21 ± 0.1	2.02 ± 0.2	6.13 ± 1.1	5.11 ± 0.1	

**Table 4:** Water vapour permeability of studied systems.

Substrate with and without the Consolidant or Coating	Water Vapour Permeability µ (/)
brick	31.9 ± 1.6
brick CF4	31.9 ± 2.4
brick PF	31.5 ± 1.5
mortar	23.8 ± 1.9
mortar CFW	23.9 ± 1.7
mortar PF	23.7 ± 2.2
